# Flywheel resistance training promotes unique muscle architectural and performance‐related adaptations in young adults

**DOI:** 10.1002/ejsc.12215

**Published:** 2024-11-05

**Authors:** Nile F. Banks, Alexander C. Berry, Emily M. Rogers, Nathaniel D. M. Jenkins

**Affiliations:** ^1^ Department of Health and Human Physiology University of Iowa Iowa City Iowa USA; ^2^ Department of Kinesiology The University of Wisconsin Madison Wisconsin USA; ^3^ School of Kinesiology Auburn University Auburn Alabama USA; ^4^ Abboud Cardiovascular Research Center University of Iowa Iowa City Iowa USA; ^5^ Fraternal Order of Eagles Diabetes Research Center University of Iowa Iowa City Iowa USA

**Keywords:** countermovement jump, isoinertial exercise, muscle hypertrophy, muscle plasticity, resistance exercise

## Abstract

The purpose of this study was to examine the skeletal muscle hypertrophic, architectural, and performance‐related adaptations in response to volume‐matched, total‐body flywheel versus traditional resistance training in a randomized, non‐exercise controlled study in physically active young adults. Thirty‐one healthy young adults (24 ± 3 y) were randomized to 10 weeks of traditional resistance training (TRT; *n* = 7F/5M), flywheel training (FWRT; *n* = 7F/4M), or a habitual activity control (CON; *n* = 5F/3M). Maximal voluntary isometric torque (MVIT), one repetition‐maximum (1RM) for the free weight squat and bench press, three repetition work maximum (3W_max_) for the flywheel squat and bench press, countermovement jump height, and broad jump distance, as well as site‐specific muscle hypertrophy, fascicle length (FL), and pennation angle, were measured. Both TRT and FWRT increased MVIT (*p* ≤ 0.021) and FFM (*p* ≤ 0.032) compared to CON. However, TRT promoted superior improvements in free weight squat and bench 1RM (*p* < 0.001), and FWRT improved flywheel 3W_max_ squat and bench (*p* < 0.001). FWRT increased the FL and cross‐sectional area of the distal VL, countermovement jump height, and broad jump distance (*p* ≤ 0.048), whereas TRT increased the pennation angle and cross‐sectional area of the proximal VL. Therefore, 10 weeks of volume‐matched, total‐body traditional, and flywheel resistance training similarly increased maximal isometric strength and fat‐free mass. However, FWRT promoted unique skeletal muscle architectural adaptations that likely contributed to region‐specific VL hypertrophy and jump performance improvements. Thus, FWRT provides a novel training stimulus that promotes architectural adaptations that support improved athletic performance in a manner that is not provided by traditional resistance exercise training.

## Highlights

- Participants using the flywheel isoinertial training device developed unique skeletal muscle adaptations in the vastus lateralis compared to the traditional resistance training group.
- The flywheel group also increased both their countermovement jump height and broad jump distance, whereas there were no improvements in the traditional resistance training group.
- The traditional training group had greater improvements in the free weight back squat and bench press compared to the flywheel group.
- Both groups improved fat‐free mass and isometric strength to a similar degree compared to the habitually exercising control group.

## 1 INTRODUCTION

Resistance training (RT) is traditionally performed utilizing isotonic exercise modalities, including free weights, cable stacks, or body weight to provide external resistance (Haff & Triplett, 2016). The prescription of isotonic RT intensity is based on concentric strength capability because concentric force production is the limiting factor in the completion of isotonic RT movements. Due to markedly greater force production capabilities during eccentric versus concentric muscle actions (Smith et al., 1998), isotonic RT modalities are limited in their ability to stress the muscle during the eccentric phase of the movement (Douglas et al., 2017). Additionally, the ability to maximally stress or overload the eccentric phase in resistance training programs is limited practically, as it typically requires an isokinetic dynamometer or manual manipulation of weight with the assistance of weight releasers or another lifter or spotter. However, flywheel devices are compact and can be used to train the entire body using common RT movements, providing an accessible means to provide an augmented eccentric stimulus in RT programs.

**FIGURE 1:**
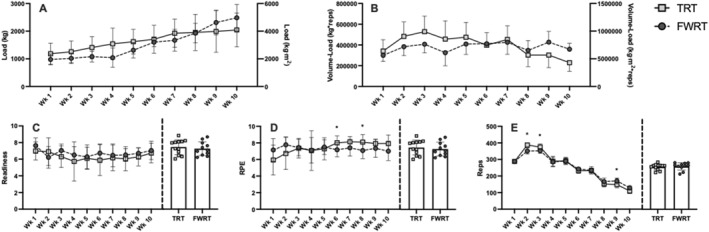
Data from training sessions held during the 10‐week intervention period for the traditional resistance training (TRT) and flywheel resistance training (FWRT) groups. Panel A: Average weekly weight lifted, expressed in kg for TRT and kg·m^2^ for FWRT; Panel B: Total (left) and average (right) weekly volume load; Panel C: Total (left) and average (right) weekly readiness, which was asked before every training session on a 0–10 scale, with 10 indicating maximal self‐perceived readiness; Panel D: Total (left) and average (right) weekly session rating of perceived exertion (RPE), which was asked immediately following each training session on a 0–10 scale with 10 representing maximal self‐perceived session exertion. Panel E: Total (left) and average (right) weekly reps completed. *, between‐group difference within the corresponding week (*p* < 0.05).

Flywheel devices were initially developed and used to resistance train astronauts during space flight (Berg et al., 1994). These devices store kinetic energy during concentric muscle actions using inertial discs that must then be resisted during the eccentric portion of the movement (see Figure 1 in (Banks et al., 2023)), providing a greater eccentric stimulus than during traditional isotonic RT. Eccentric overload training has been shown to elicit specific physiological responses that may ultimately promote different adaptations than traditional RT (Douglas et al., 2017; Franchi et al., 2014; Friedmann‐Bette et al., 2010; Gehlert et al., 2015). For example, the high mechanical stress imposed by eccentric overload promotes distinct molecular that include greater upregulation of mitogen‐activated protein kinases such as the stress‐activated protein kinase p38 (MAPK(p38)) (Franchi et al., 2014; Gehlert et al., 2015; Wretman et al., 2001) and in the expression of genes functionally associated with protein synthesis, the early growth response, and structural remodeling (Kostek et al., 2007). Subsequently, eccentric overload has also been linked with different morphological and architectural adaptations, such as a potentially greater propensity for type IIa and IIx fiber hypertrophy and increased fascicle length (FL) than traditional RT (Franchi et al., 2014; Friedmann‐Bette et al., 2010). While flywheel devices do not necessarily provide eccentric overload (Munoz‐Lopez et al., 2021), the limitation of conscious, eccentric braking to a portion of the eccentric phase of the movement to resist the kinetic energy of the flywheel augments the eccentric stimulus compared to traditional training. Therefore, the ability of flywheel devices to provide an augmented eccentric stimulus while simultaneously training the concentric portion of a movement may prove to be superior to traditional, isotonic RT methods, particularly regarding muscle morphological and architectural adaptations and perhaps performance‐related movements that require a high rate of force development like jumping.

Multiple studies have compared lower‐body RT programs using flywheel‐based or traditional RT, primarily reporting similar increases in isometric and dynamic strength (Caruso et al., 2005; Maroto‐Izquierdo, García‐López, Fernandez‐Gonzalo, et al., 2017; Norrbrand et al., 2008, 2010). There is also initial evidence that flywheel training may augment countermovement jump performance (de et al., 2015; Maroto‐Izquierdo, García‐López, & de Paz, 2017; Stojanović et al., 2021) and promote architectural adaptations such as increased FL (Seynnes et al., 2007). However, it is unclear if these adaptations are unique relative to the effects of traditional RT. Notably, FL has been positively associated with contractile velocity (Stasinaki et al., 2019; Timmins et al., 2016), and it is, therefore, conceivable that flywheel training could improve countermovement jump performance by promoting unique architectural adaptations, but to our knowledge, this has yet to be directly assessed. Thus, well‐controlled, randomized studies are still necessary to understand whether flywheel training promotes unique morphological and architectural adaptations compared to traditional, isotonic RT and if unique performance‐related improvements accompany these adaptations.

Prior studies comparing traditional isotonic to flywheel RT have included methodological choices that have limited internal or external validity. Limitations include nonmatching exercise selections (de et al., 2015), suboptimal body composition measures (Caruso et al., 2005), single joint exercise training (Norrbrand et al., 2008, 2010), and the lack of a true non‐exercise control group (Caruso et al., 2005; Norrbrand et al., 2008; Norrbrand et al., 2010; de et al., 2015; Maroto‐Izquierdo, García‐López, & de Paz, 2017). Further, a recent meta‐analysis (Maroto‐Izquierdo, García‐López, Fernandez‐Gonzalo, et al., 2017) that compared flywheel versus traditional RT adaptations included four (of the total 9) studies that did not compare adaptations in response to flywheel versus resistance training but rather flywheel versus a non‐training control group. To bridge the gap between mechanistic and isolated findings and practice, well‐constructed training studies are desperately needed. To date, no randomized, controlled trials have compared a volume‐ and exercise‐matched resistance training program using a flywheel device versus traditional isotonic resistance training using free weights and cable stacks on measures of strength and hypertrophy. In addition, few studies have attempted to evaluate if flywheel and traditional training promote different muscle architectural adaptations, such as in fascicle length or pennation angle. Therefore, the purpose of the current study was to determine the skeletal muscle hypertrophic, architectural, and performance‐related adaptations in response to a volume‐matched, total‐body flywheel versus traditional resistance training program in a randomized, non‐exercise controlled study in physically active young adults.

## 2 METHODS

### 2.1 Participants

Forty‐three healthy, physically active, young adult males and females completed a screening visit and were deemed eligible. Eligible participants were randomized into either a traditional training (TRT), flywheel training (FWRT), or a control group (CON). Following the screening process, 12 participants dropped out of the study for the following reasons: scheduling conflicts (*n* = 5), low adherence to protocol (*n* = 3), disliked group assignment (*n* = 1), unrelated injury (*n* = 1), illness (*n* = 1), and lost to follow‐up (*n* = 1). Of the 12 participants who dropped, only five completed pre‐testing, and none began the intervention. Therefore, 31 individuals (TRT, *n* = 12 [7 F]; FWRT, *n* = 11 [7 F]; CON, *n* = 8 [5 F]) completed this study (Table 1). Before enrollment, participants completed an informed consent form, health history questionnaire, and the physical activity readiness questionnaire (PAR‐Q+). To be eligible, participants must have been 18–30 years old, had a body mass index of 18.5–39.9 kg/m^2^, been healthy according to self‐reported health history, and be ready to begin an exercise training program according to the PAR‐Q+. Participants were recruited using IRB‐approved emails and the university mass email system, as well as by word of mouth. All study procedures and documents complied with the Declaration of Helsinki and were approved by the University's Institutional Review Board for the protection of human subjects (IRB Approval #: 202008153). All participants consented to participation by signing an informed consent form, which explained the study's nature, benefits, and risks before participation.

**TABLE 1:** Baseline participant characteristics.

	TRT (*n* = 12; 5M/7F)	FWRT (*n* = 11; 4M/7F)	CON (*n* = 8; 3M/5F)
Age (y)	25.75 (3.0)	22.73 (3.1)	21.38 (2.2)
Height (cm)	1.68 (0.1)	1.72 (0.1)	1.69 (0.1)
Weight (kg)	74.5 (14.3)	72.6 (17.9)	68.0 (8.9)
BMI (kg/m^2^)	26.24 (4.57)	24.36 (4.02)	23.82 (3.19)
Currently lifting^a^ (%)	50%	46%	63%
RT experience (y)	4.3 (3.4)	3.2 (2.7)	2.6 (2.2)
Squat frequency^b^ (sessions/week)	0.67 (0.5)	0.7 (0.7)	0.9 (0.5)
Deadlift frequency^b^ (sessions/week)	0.33 (0.5)	0.9 (0.5)	0.67 (0.5)
Bench frequency^b^ (sessions/week)	0.67 (0.5)	0.6 (0.6)	1.2 (0.8)

*Note*: All data are displayed as mean (SD).

Abbreviations: BMI, body mass index; CON, control; FWRT, flywheel resistance training; TRT, traditional resistance training.

^a^ Participants were determined to be currently lifting if they self‐reported to be regularly engaging in resistance training (RT) during the past 6 months.

^b^ Self‐reported frequency during the previous 6 months in only those participants who reported to be regularly engaging in RT.

### 2.2 Power calculation

Prior sample size estimates were obtained using G*Power (v2, Germany). We powered to detect a medium within between interaction effect of 0.25 at 1 − *β* (power) of 0.8, assuming three experimental groups (FWRT vs. TRT vs. CON) and two measurements (pre‐vs. post‐training). A correlation of 0.7 between repeated measurements was assumed based conservatively on interclass correlation coefficients for ultrasound‐based measurements of muscle size and both isometric and dynamic muscle strength for our lab (Jenkins et al., 2015). We also calculated the sample size necessary to observe a between‐group difference in muscle thickness and vertical jump performance for flywheel versus traditional training at the post‐training timepoint (Maroto‐Izquierdo, García‐López, & de Paz, 2017). Based on these calculations, 9 participants were needed in each group, or 12 participants to each group when accounting for a potential dropout rate of 25%.

### 2.3 Experimental design

Each participant visited the laboratory for four experimental visits, two of which took place during the week immediately before and two which took place during the week after the 10‐week intervention period. Before all experimental visits, participants were asked to abstain from caffeine for 12 h. Body composition was assessed at the first experimental visit following a fast of at least 6, whereas skeletal muscle ultrasound, isometric strength testing, jump testing, and strength testing were performed during the second experimental visit in that order. A washout period of at least 48 h separated each visit at pre‐ and post‐testing. The 10‐week intervention period began 3–7 days following the second experimental visit, and the first experimental visit at post‐testing was held 3–7 days following the final training session for TRT and FWRT.

### 2.4 Resistance training protocol

A detailed description of the resistance training program is shown in Table 2. Participants who were randomized into TRT or FWRT partook in a 10‐week, volume‐matched, periodized, total‐body resistance training program performed on a flywheel device (Exerfly Training Platform; Exerfly Sport Limited, New Zealand) or with free weights and cable stacks. The flywheel device used in the current study had two attachment points along the flywheel to allow for a barbell to be attached, as well as a bench attachment, which allowed identical form to be used for the barbell squat, bench press, row, and deadlift in FWRT as in TRT (Banks et al., 2024). The program consisted of three workouts per week utilizing both compound and assistance movements with three sets of four or five exercises performed in each workout. The load progressed from ∼12 repetition maximum (RM) to 4RM in the four primary compound movements throughout the training program. Following the first week, which served as an introductory week, during the 3^rd^ set for the squat, bench press, deadlift, and row, participants performed as many repetitions as possible until technical failure, but not more than four repetitions than the exercise prescription for that day. For example, if the exercise prescription was 4RM, the participant was allowed to complete up to (but no more than) 8 repetitions, even if they could complete additional repetitions before achieving technical failure. The load for the following session was then increased by 5%, 7.5%, or 10% if the participant achieved 1, 2, or 3–4 repetitions greater than prescribed, respectively. For the FWRT group, since device resistance can be reduced by a decrease in movement speed, the work (J) that was produced during each repetition was used to prescribe load. Specifically, work for each repetition was monitored in real time on an external computer, providing live feedback for each individual repetition and the total work completed for each set. Failure in the 3rd set for FWRT was then determined to occur either when participants reached technical failure or when two consecutive repetitions occurred that were below 20% of the average work produced during the 2nd set. Participants were asked to refrain from any additional resistance training during the entire study period. Additionally, they were asked to attempt to maintain current physical activity habits outside of the study, which was confirmed via completion of the IPAQ throughout the intervention. Individuals in the CON group completed all aspects of the study outside of the structured training during the 10‐week intervention period.

**TABLE 2:** The 10‐week resistance training protocol using either a flywheel training device or traditional gravity‐based weights.

Lift 1	Lift 2	Lift 3	Week 1	Weeks 2–3	Weeks 4–5	Weeks 6–8	Week 9	Week 10
Squat	Squat	Squat	3 × 8	3 × 12 RM	3 × 10 RM	3 × 8 RM	3 × 6 RM	3 × 4 RM
Bench press	Bench press	Bench press	3 × 8	3 × 12 RM	3 × 10 RM	3 × 8 RM	3 × 6 RM	3 × 4 RM
Deadlift	Deadlift	Deadlift	3 × 8	3 × 12 RM	3 × 10 RM	3 × 8 RM	3 × 6 RM	3 × 4 RM
Row	Row	Row	3 × 8	3 × 12 RM	3 × 10 RM	3 × 8 RM	3 × 6 RM	3 × 4 RM
Bicep curl	Triceps extension	Glute bridge	‐	‐	‐	3 × 12 RM	3 × 10 RM	3 × 8 RM

Abbreviation: RM, repetition maximum.

### 2.5 Control group

CON completed all aspects of the study exercise except for the resistance training intervention and were instead instructed to maintain their current dietary and physical activity habits. Thus, CON participants completed baseline and post‐intervention experimental testing, weekly surveys, and recorded dietary intake.

### 2.6 Skeletal muscle ultrasound

Ultrasound images of the knee extensors and flexors, elbow extensors and flexors, and plantar flexors were obtained by the same researcher using a portable brightness mode ultrasound imaging device (GE Logiq P9, USA) and a 12‐MHz multifrequency linear‐array probe (12L‐RS, General Electric, USA). While participants were lying on their side with an ∼80° knee joint angle, a distance of 33%, 50%, and 66% from the anterior superior iliac spine and the lateral epicondyle was marked and used for the measurement of the proximal (VL_PROX_), middle (VL_MID_), and distal portions of the vastus lateralis (VL_DIST_). A distance of 50% from the anterior iliac spine to the superior patella was marked and was used for the measurement of the rectus femoris (RF). With participants in a prone position, 50% of the distance between the infraglenoid tubercle of the scapula to the posterior olecranon was used as the measurement site for the belly of the triceps brachii long head, and 33% of the distance from the medial and lateral condyle and the posterior surface of the calcaneus were used for the medial and lateral gastrocnemius, respectively. With participants in a supine position, 66% of the distance from the supraglenoid tubercle of the scapula to the radial tuberosity was used as the measurement site for the biceps brachii long head.

A generous amount of water‐soluble transmission gel was applied over each area and on the surface of the ultrasound probe, and minimal pressure was applied to the ultrasound probe during image capture to ensure high‐quality imaging with minimal tissue compression. Frequency, gain, dynamic range, and image depth were held constant for all images. Three still sagittal ultrasound images were taken over the belly of the biceps brachii long head, triceps brachii long head, medial gastrocnemius, and lateral gastrocnemius. While participants were lying on their side with an ∼80° knee bend, extended field of view was used to collect three cross‐sectional ultrasound scans at the level of the RF, VL_PROX_, VL_MID_, and VL_DIST_, and three scans from the lateral epicondyle to the trochanter major with the ultrasound probe held parallel to the VL fascicles to determine both FL and the pennation angle (Noorkoiv et al., 2010).

All images were then analyzed offline by a blinded researcher using image analysis software (ImageJ, version 1.51i). For the quantification of the muscle cross‐sectional area (mCSA) from panoramic transverse images, the muscle of interest was measured by including as much muscle as possible without including surrounding fascia (Jenkins et al., 2015). The distance between the superficial and deep aponeurosis was used to quantify muscle thickness (MT) from still, sagittal images (Jenkins et al., 2017a). The average of all three images at each site was then used for the analysis of mCSA (cm^2^), MT (cm), FL (cm), and pennation angle (degrees).

### 2.7 Body composition

Participants' body composition was assessed using a three‐compartment model, including total‐body water derived from bioelectrical impedance analysis (seca 514, seca, USA) and body density via BodPod (COSMED, USA). The following formulas were used to assess fat mass (FM) and fat‐free mass (FFM), where body density was measured in kg/L, total‐body water in L, and body mass in kg (Siri, 1993):

FM=BodyMass×(BF%÷100)

FFM=BodyMass−FM

### 2.8 Dynamic muscle strength

Dynamic muscle strength was assessed via the estimated one repetition maximum (1RM) for the free weight squat and bench press and work produced during three repetitions (3W_max_) for the squat and bench press on the flywheel device. During the pre‐ and post‐training testing sessions, 1–3 RM testing was conducted based on the National Strength and Conditioning Association guidelines (Haff & Triplett, 2016). To acquire flywheel squat and bench press 3W_max_ work (J), participants were familiarized with the flywheel device and taught the proper technique for each movement at a conservative weight. Participants were instructed to complete the concentric portion of each movement as quickly as possible before transitioning into maximal resistance immediately upon entering the eccentric phase to deplete the kinetic energy of the flywheel device within the first third of the eccentric phase. Weight was then increased until participants could no longer increase the work produced during the three repetitions. Work for each rep was obtained and recorded using the Exerfly App (Exerfly Training Platform; Exerfly Sport Limited, New Zealand).

### 2.9 Isometric muscle strength

Leg extension MVIT was assessed on a custom‐built isometric dynamometer (COR1, OT Bioelettronica, Italy). Participants were seated in a specialized chair with restraining straps across the shoulders and hips, and their legs flexed so that it was at a 120° angle (180° representing full extension). A strap was attached 3–4 cm proximal to the medial malleolus and wrapped around a wooden leg rest attached to a load cell. Participants completed three submaximal warm‐up muscle actions at increasing intensities followed by two separate 4–5 s maximal voluntary isometric contractions (MVIT) with 1 min of rest provided between each attempt. The greatest torque produced during a 1‐s period from the maximal attempts represented the MVIT.

### 2.10 Jump performance

Countermovement jump and broad jump performance were assessed per the NSCA testing guidelines (Haff & Triplett, 2016). Countermovement jump height was assessed using a stand‐alone jump measurement tester (Tandem Sport Vertical Challenger, Tandem Sport, USA). For the broad jump, participants were instructed to jump as far as possible over a strip of measuring tape taped to the floor to assess jump distance. Participants were required to take at least three attempts for all jump tests and were provided more attempts if jump height increased on subsequent jumps until a maximal value was achieved.

### 2.11 Lifestyle controls

During the study, all participants were asked to refrain from all other forms of exercise outside of the study. Other than the request to maintain typical dietary habits, no dietary advice was given. However, participants were asked to log their food on a commercially available mobile app (MyFitnessPal, USA) to verify compliance during the entire study period. In order to track physical activity levels during the study period, participants completed the International Physical Activity Questionnaire (IPAQ) at the end of each week. Lastly, participants completed the multicomponent training distress scale (MCTDS) each week in order to assess depression, vigor, physical symptoms, sleep disturbances, stress, and fatigue (Main et al., 2016).

### 2.12 Statistics

Multiple independent two‐way mixed linear models (group × visit) were run to determine the impact of training modality on all strength, body composition, muscle size and architecture, and jump performance outcomes. For MCTDS data, exercise groups displayed uniform responses and were thus combined to be compared to the control group (EX vs. CON). Two‐way mixed linear models (group × time) were utilized to examine MCTDS data between EX and CON each week. One‐way linear models were used to compare groups for physical activity and dietary data. Independent samples *t*‐tests were utilized to compare repetitions completed and the percent increase in weight, readiness, and RPE between TRT and FWRT during the intervention period. When significant group × visit interactions, group main effects, or visit main effects occurred, one‐way linear models and Tukey‐adjusted post hoc analyses were performed. Effect sizes were determined using Cohen's d. Pearson's product correlations were used to assess the relationship between changes in FL and changes in countermovement jump height and broad jump distance. Data are reported as mean (lower–upper 95% CI) unless denoted otherwise, and significance was set at *p* ≤ 0.05. Statistical analyses were performed using R (v. 4.1.2), and figures were created using GraphPad Prism (v. 8.4.3).

## 3 RESULTS

### 3.1 Training data

Training adherence, dietary intake, and physical activity level data have been previously published and were similar between groups (Banks et al., 2024). Adherence to the exercise intervention was 93.3% and 94.9% in TRT and FWRT. There was a significant group × time interaction for RPE (*p* < 0.001) and the total number of repetitions completed (*p* < 0.001) but not for readiness (*p* = 0.47) during the training period. Specifically, RPE was significantly greater in weeks 6 and 8 in TRT compared to FWRT, but there were no other between‐group differences during any other week. Consequently, the average RPE across the entire exercise intervention did not differ significantly between groups (Figure 1D). The total number of repetitions completed was significantly greater in weeks 2 and 3 in TRT compared to FWRT and higher in FWRT during week 9; however, the average repetitions completed across the entire 10‐week intervention period were similar for the TRT and FWRT (Figure 1E).

### 3.2 Muscle size

**FIGURE 2:**
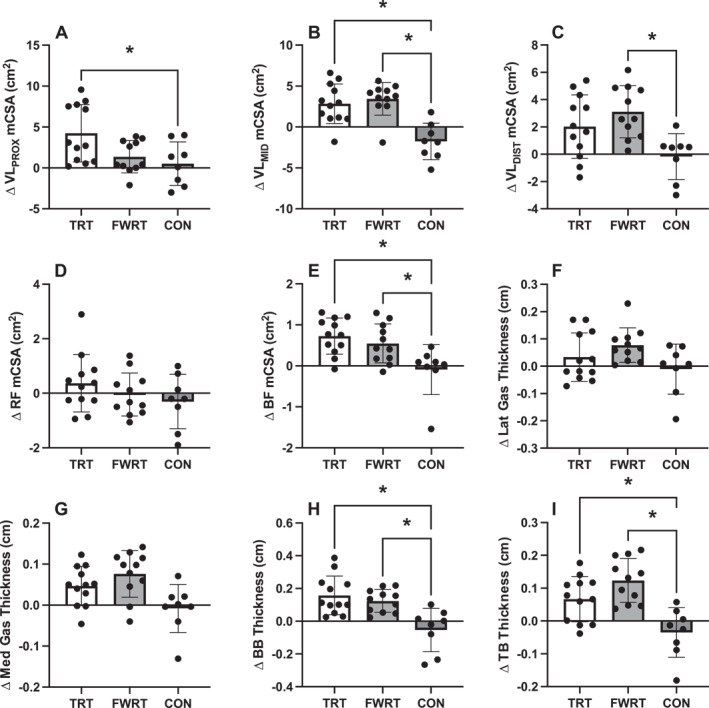
Group changes in individual muscle size measured using ultrasonography before and after 10 weeks of traditional resistance training (TRT), flywheel resistance training (FWRT), or a control period (CON); * = *p* < 0.05 when comparing the pre‐ to post‐training within‐group changes between groups using a one‐way linear model; BB, biceps brachii; BF, biceps femoris; Gas, gastrocnemius; Lat, lateral; mCSA, muscle cross‐sectional area; Med, medial; RF, rectus femoris; TB, triceps brachii; VL_DIST_, the distal portion of the vastus lateralis; VL_MID_, the middle portion of the vastus lateralis; VL_PROX_, the proximal portion of the vastus lateralis.

When examining local muscle size changes measured using ultrasonography (Figure 2), there was a significant group × visit interaction for VL_PROX_ mCSA (*p* = 0.014), VL_MID_ mCSA (*p* < 0.001), VL_DIST_ mCSA (*p* = 0.006), biceps femoris mCSA (*p* = 0.004), biceps brachii thickness (*p* < 0.001), and triceps brachii thickness (*p* < 0.001) but not rectus femoris mCSA (*p* = 0.30), medial gastrocnemius thickness (*p* = 0.09), nor lateral gastrocnemius thickness (*p* = 0.20). Specifically, TRT (+3.71 cm^2^ [0.52–6.90], *d* = 1.31, *p* = 0.02) but not FWRT (+0.85 cm^2^ [−2.40–4.10], *d* = 0.30, *p* = 0.8) elicited a greater increase in VL_PROX_ mCSA compared to CON, but the increase observed in TRT was not significantly different relative to FWRT (+2.861 cm^2^ [−0.06–5.78], *d* = 1.01, *p* = 0.056). Both TRT (+4.6 cm^2^ [2.08–7.12], *d* = 2.06, *p* < 0.001) and FWRT (+5.21 cm^2^ [2.64–7.77], *d* = 2.33, *p* < 0.001) elicited greater increases in VL_MID_ mCSA than CON, and this increase was not different for TRT versus FWRT (+0.604 cm^2^ [−1.70–2.91], *d* = 0.27, *p* = 0.80). FWRT (+3.29 cm^2^ [0.95–5.62], *d* = 1.62, *p* = 0.005) but not TRT (+2.2 cm^2^ (2.20 [−0.10–4.49], *d* = 1.08, *p* = 0.06)) elicited a greater increase in VL_DIST_ mCSA compared to CON. However, the increase in VL_DIST_ mCSA elicited by FWRT was not different relative to TRT (+1.092 cm^2^ [−1.01–3.19] *d* = 0.54, *p* = 0.41). Both TRT (+0.82 cm^2^ [0.25–1.38], *d* = 1.64, *p* = 0.004) and FWRT (+0.64 cm^2^ [0.06–1.21], *d* = 1.28, *p* = 0.027) elicited greater increases in biceps femoris mCSA compared to CON, and the increases in TRT and FWRT were not different (+0.179 cm^2^ [−0.34–0.70] *d* = 0.36, *p* = 0.67). Both TRT (+0.21 cm [0.09–0.33], *d* = 1.96, *p* < 0.001) and FWRT (+0.18 cm [0.05–0.30], *d* = 1.65, *p* = 0.004) elicited greater increases in biceps brachii thickness compared to CON, and the increases in TRT and FWRT were not different (+0.034 cm [−0.08–0.15], *d* = 0.31, *p* = 0.74). Lastly, both TRT (+0.1 cm [0.02–0.18], *d* = 1.46, *p* = 0.01) and FWRT (+0.16 [0.16–0.08], *d* = 2.27, *p* = 0.001) elicited greater increases in triceps brachii thickness compared to CON, while there was no difference between FWRT and TRT (+0.057 cm [−0.02–0.13], *d* = 0.81, *p* = 0.15) (Figure 2). Among the variables where no significant interaction was observed, there were also no significant group or visit main effects (all *p* ≥ 0.28), except for medial gastrocnemius thickness, which increased from pre‐to post‐intervention independent of the group (+0.05 cm, *p* = 0.006).

### 3.3 Muscle architecture

**FIGURE 3:**
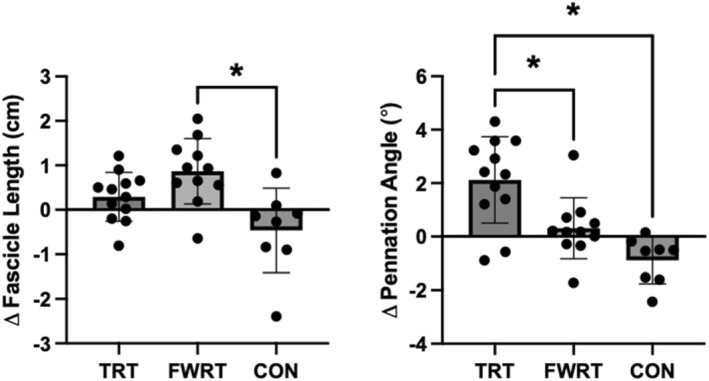
Group changes in fascicle length and pennation angle derived from ultrasonography before and after either 10 weeks of traditional resistance training (TRT), flywheel resistance training (FWRT), or a habitual activity, non‐exercise control period (CON); * = *p* < 0.05 when comparing the pre‐ to post‐training within‐group changes between groups using a one‐way linear model.

All muscle architecture data are displayed in Figure 3. There was a significant group × visit interaction for FL (*p* = 0.002), where FWRT elicited significantly greater increases in FL compared to CON (+1.33 cm [0.49–2.17], *d* = 1.82, *p* = 0.002) but not TRT (+0.58 cm [−0.18–1.33], *d* = 0.79, *p* = 0.16). However, TRT did not elicit significant improvements relative to CON (+0.75 cm [−0.07–1.58], *d* = 1.03, *p* = 0.08). There was also a significant group × visit interaction for the pennation angle (*p* < 0.001), where TRT elicited greater increases compared to both CON (+3.01° [1.54–4.47], *d* = 2.32, *p* < 0.001) and FWRT (+1.81° [0.47–3.15], *d* = 1.39, *p* = 0.007). However, FWRT did not elicit significant improvements relative to CON (+1.2° [−0.29–2.69], *d* = 0.92, *p* = 0.13).

### 3.4 Body composition

All whole‐body body composition data are displayed in Table 3. There was a significant group × visit interaction for FFM (*p* = 0.008), where both TRT (+1.72 kg [0.41–3.03], *d* = 1.48, *p* = 0.008) and FWRT (+1.44 kg [0.106–2.775], *d* = 1.24, *p* = 0.032) elicited greater FFM increases than CON, and there was no difference in the improvement for TRT versus FWRT (+0.28 kg [−0.92–1.48], *d* = 0.24, *p* = 0.83). There was no group × visit interaction nor main effect for either group or visit for FM (*p* = 0.30–0.76).

**TABLE 3:** Body composition, strength, and jump performance data before and after the intervention period in the TRT, FWRT, and CON groups.

	TRT (*n* = 12; 5M/7F)		FWRT (*n* = 11; 4M/7F)		CON (*n* = 8; 3M/5F)		*p*		
	Pre	Post	Pre	Post	Pre	Post	TRT versus CON	FWRT versus CON	TRT versus FWRT
Body weight (kg)	74.5 (14.3)	75.5 (14.6)	72.6 (17.9)	73.4 (17.2)	68.0 (8.9)	68.1 (8.4)	0.451	0.572	0.978
FFM (kg)	52.7 (8.9)	54.2 (8.9)	51.3 (9.7)	52.5 (9.3)	50.8 (8.2)	50.7 (7.4)	0.008*	0.032*	0.834
FM (kg)	21.6 (8.6)	21.1 (8.4)	21.0 (11.3)	20.7 (10.8)	16.9 (8.3)	17.3 (8.4)	0.519	0.647	0.979
BF(%)	28.4 (7.8)	27.3 (7.1)	27.9 (8.9)	27.1 (8.5)	24.6 (10.3)	25.0 (9.9)	0.217	0.373	0.932
BB squat 1RM (kg)	162.2 (47.0)	249.3 (56.3)	137.7 (35.9)	181.5 (47.3)	143.7 (62.6)	151.5 (65.8)	<0.001*	0.002*	<0.001*
BB bench 1RM (kg)	112.3 (49.9)	136.7 (54.5)	92.1 (37.1)	100.9 (40.1)	98.3 (53.5)	100.9 (61.0)	<0.001*	0.251	<0.001*
FW squat 3W_max_ (J)	2320.1 (1409.7)	2846.3 (1685.8)	1757.3 (970.8)	3062.9 (1676.1)	2400.5 (2112.4)	2244.8 (1680.5)	0.237	0.004*	0.112
FW bench 3W_max_ (J)	1167.3 (693.8)	1344.1 (618.7)	849.8 (458.0)	1256.4 (578.4)	970.9 (739.1)	951.5 (641.3)	0.256	<0.001*	0.117
MVIT (Nm)	387.7 (1.7)	432.0 (3.3)	432.6 (3.5)	473.2 (3.6)	350.8 (1.8)	318.1 (2.0)	0.014*	0.023*	0.984
CMJ (cm)	43.8 (8.3)	45.3 (8.3)	42.7 (9.2)	45.7 (9.9)	43.2 (13.6)	43.8 (12.4)	0.639	0.048*	0.193
BJ (cm)	452.8 (95.5)	484.3 (80.3)	426.3 (82.2)	479.8 (74.1)	466.7 (99.0)	472.8 (86.0)	0.182	0.007*	0.216

*Note*: All data are displayed as mean (SD).

Abbreviations: 1RM, one repetition maximum; 3W_max_, the maximal amount of work produced in three reps; Abs, absolute; BB, barbell; BJ, broad jump; CMJ, counter movement jump; CON, control; F, female; FFM, fat free mass; FM, fat mass; FWRT, flywheel resistance training; M, male; MVIT, maximal voluntary isometric torque; TRT, traditional resistance training.

* = *p* < 0.05 comparing between‐group group changes from pre‐to post‐testing.

### 3.5 Strength

All strength data are displayed in Table 3. There was a significant group × visit interaction for maximal voluntary isometric torque (MVIT; *p* = 0.011), free weight squat 1RM (*p* < 0.001), free weight bench 1RM (*p* < 0.001), flywheel squat 3W_max_ (*p* = 0.006), and flywheel bench 3W_max_ (*p* = 0.006). Specifically, both TRT (+78.56 Nm [9.28–139.45], *d* = 1.42, *p* = 0.014) and FWRT (+56.07 Nm [−27.92–140.08], *d* = 1.35, *p* = 0.023) increased MVIT more than CON, and there was no difference in the improvement in MVIT between TRT and FWRT (−4.19 Nm [−55.76–64.14], *d* = 0.07, *p* = 0.97). However, TRT increased free weight squat 1RM more than both CON (+79.32 kg [56.02–102.62], *d* = 3.84, *p* < 0.001) and FWRT (+43.28 kg [21.97–64.59], *d* = 2.1, *p* < 0.001), while FWRT improved more than CON (+36.04 kg [12.32–59.76], *d* = 1.75, *p* = 0.002). Similarly, TRT increased free weight bench 1RM more than both CON (+21.79 kg [12.57–31.03], *d* = 2.67, *p* < 0.001) and FWRT (+15.62 kg [7.18–24.06], *d* = 1.91, *p* < 0.001), while FWRT did not improve more than CON (+6.18 kg [−3.22–15.58], *d* = 0.76, *p* = 0.25). On the other hand, FWRT improved flywheel squat 3W_max_ compared to CON (+1461.33 J [429.10–2493.57], *d* = 1.63, *p* = 0.004) but not TRT (+779.37 J [‐147.93–1706.67], *d* = 0.87, *p* = 0.11), whereas TRT did not significantly improve flywheel squat 3W_max_ relative to CON (+681.96 J [−332.0–1695.93], *d* = 0.76, *p* = 0.24). Similarly, FWRT improved flywheel bench 3W_max_ compared to CON (+426.1 J [125.11–727.10], *d* = 1.63, *p* = 0.004) but not TRT (+229.85 J [−46.36–506.07], *d* = 0.88, *p* = 0.12), while TRT did not significantly improve flywheel bench 3W_max_ relative to CON (+196.25 J [−104.75–497.25], *d* = 0.75, *p* = 0.26).

### 3.6 Jump performance

All jump performance data are displayed in Table 3. There was a significant group × visit interaction for broad (*p* = 0.009) and countermovement jump performance (*p* = 0.049). Specifically, FWRT elicited greater improvements in broad jump distance compared to CON (+47.54 cm [12.21–82.86], *d* = 1.55, *p* = 0.007) but not TRT (+22.03 cm [−9.70–53.77], *d* = 0.72, *p* = 0.22). Further, TRT did not improve broad jump distance significantly when compared to CON (+25.51 cm [−9.20–60.21], *d* = 0.83, *p* = 0.18). FWRT also elicited greater improvements in countermovement jump height than CON (+2.37 cm [0.02–4.71], *d* = 1.16, *p* = 0.048) but not TRT (+1.52 cm [−0.59–3.63], *d* = 0.75, *p* = 0.19). Again, TRT did not improve countermovement jump height significantly when compared to CON (+0.85 cm [−1.46–3.15], *d* = 0.42, *p* = 0.64).

### 3.7 Lifestyle controls

**FIGURE 4:**
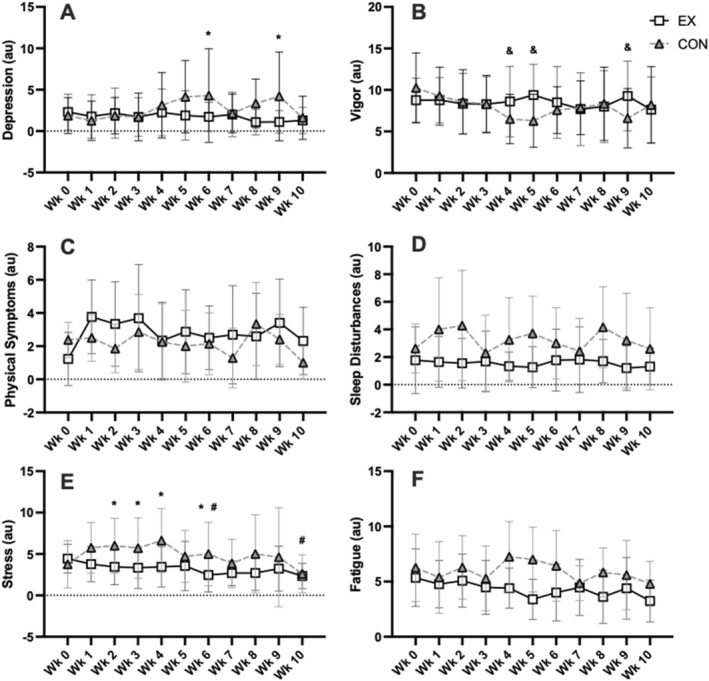
The multicomponent training distress scale was utilized to assess weekly levels of depression (Panel A), vigor (Panel B), physical symptoms (Panel C), sleep disturbances (Panel D), stress (Panel E), and fatigue (Panel F). *, significant group difference; &, significant difference in CON compared to Week 0; #, significant difference in EX compared to week 0.

There were no significant differences in calories (*p* = 0.41), carbohydrates (*p* = 0.35), fat (*p* = 0.58), protein (*p* = 0.95), or MET/min/week (*p* = 0.30). There was a significant group × time interaction for depression (*p* = 0.015), vigor (*p* = 0.041), and stress (*p* = 0.039) (Figure 4). While there were no group × time interactions for physical symptoms (*p* = 0.09), Sleep (*p* = 0.37), or fatigue (*p* = 0.22), there were significant group main effects for sleep and fatigue. Specifically, sleep disturbances (−1.84 au; *p* = 0.023) and fatigue (−1.67 au; *p* = 0.034) were significantly lower in EX than CON. There was no group main effect for physical symptoms (*p* = 0.33).

### 3.8 Correlation analyses

There was a significant (*r* = 0.49; *p* = 0.005) and near‐significant (*r* = 0.35; *p* = 0.054) correlation between change in FL and change in broad jump and countermovement jump performance, respectively.

## 4 DISCUSSION

This is the first study to examine the effects of whole‐body, progressive, isoinertial flywheel versus traditional resistance exercise training on skeletal muscle function, size, and architecture in a non‐exercise controlled, randomized study. Our findings indicate that TRT and FWRT elicit similar improvements in isometric strength but strength improvements that are specific to the modality used in training. Further, both TRT and FWRT induced significant increases in whole‐body fat‐free mass and generally promoted a similar magnitude of muscle hypertrophy. However, our findings also indicated that TRT and FWRT might elicit region‐specific hypertrophy of the VL with TRT increasing the mCSA of VL_PROX_ and FWRT increasing the mCSA of VL_DIST_. Furthermore, whereas TRT significantly increased the pennation angle, FWRT significantly increased FL. Interestingly, FWRT also elicited significant improvements in both countermovement jump and broad jump performance, and improvements in jump performance were associated with FL increases. Thus, our findings appear to indicate that progressive, isoinertial flywheel training promotes unique muscle architectural adaptations that are associated with improved muscular performance in young adults.

This study is among the first to examine the effects of progressive, whole‐body TRT versus FWRT on whole‐body fat‐free mass and site‐specific muscle hypertrophy. We observed similar TRT‐ and FWRT‐induced increases in whole‐body FFM in the current study. Further, both TRT and FWRT elicited similar increases in individual muscle size for the biceps femoris, biceps brachii, triceps brachii long head, and the middle site of the VL. However, FWRT increased the mCSA of the distal VL, while TRT increased the mCSA of the proximal VL (Figure 2). A few prior studies have suggested that FWRT may promote greater muscle hypertrophy than TRT (Maroto‐Izquierdo et al., 2017b, 2022; Norrbrand et al., 2008). Maroto‐Izquierdo et al. reported that 6 weeks of leg press FWRT promoted significantly greater increases in proximal, middle, and distal VL muscle thickness compared to TRT (Maroto‐Izquierdo, García‐López, & de Paz, 2017). In a different study, Maroto‐Izquierdo et al. also reported that 6 weeks of shoulder FWRT promoted greater anterior and medial deltoid hypertrophy than pneumatic resistance training (Maroto‐Izquierdo et al., 2022). Norrbrand et al. reported a 6.2% versus a 3.0% increase in whole quadriceps muscle volume measured using MRI following 12 sessions of leg extension FWRT versus TRT, but this difference was not statistically significant (Norrbrand et al., 2008). It is unclear why the current study did not agree with these studies, although methodological differences such as the whole‐body training program, length of training, and training status of the participants may explain the differences. For example, the 10‐week training program used herein required participants to complete 2–2.5 times the number of training sessions (i.e., 30 vs. 12–15 sessions) as these aforementioned studies (Maroto‐Izquierdo et al., 2017b, 2022; Norrbrand et al., 2008). It has also been cautioned that edema may contribute to estimates of muscle size, particularly in response to short‐term, unaccustomed resistance training and especially where eccentric actions are emphasized (Damas et al., 2016; Jenkins et al., 2017b; Stock et al., 2017). Thus, it is plausible that the findings of these prior investigations may be inadvertently contaminated by skeletal muscle edema (Damas et al., 2016), particularly where post‐testing was performed within 24–48 h of the final training session. It is notable that in the present study, post‐testing assessments of muscle size were performed at least 72 h after the final training session, and investigators remained blinded to the group assignment and measurement timepoint to avoid biasing measurements. Finally, as previously described by Norrbrand et al. (2008) and Franchi et al. (2017), the current body of evidence does not support the superiority of constant concentric–eccentric versus eccentric overload (or eccentric vs. concentric) resistance exercise for promoting skeletal muscle hypertrophy. For example, the findings of studies that have compared work‐matched maximal eccentric versus maximal concentric isokinetic resistance exercise training on hypertrophy are equivocal (Maeo et al., 2018; Moore et al., 2012). Moore et al. (2012) reported similar increases in biceps brachii mCSA, but Maeo et al. (2018) reported greater increases in quadriceps femoris muscle volume following eccentric versus concentric training. Moreover, Unlu et al. reported similar 4%–10%, 6%–8%, and 7% increases in quadriceps femoris muscle volume in response to 12 weeks of eccentric‐only, concentric‐only, and concentric–eccentric isotonic training (Ünlü et al., 2020). Thus, our findings seem to support the prevailing evidence regarding eccentric versus concentric or concentric–eccentric resistance training (Franchi et al., 2017) and indicate that when volume (or work) and load‐matched, FWRT, and TRT promote similar muscle hypertrophy. However, our data also indicate that FWRT and TRT promote region‐specific VL muscle hypertrophy, whereby FWRT promotes hypertrophy more distally, and TRT seems to promote hypertrophy more proximally.

In the current study, only FWRT induced significant increases in FL compared to CON, whereas TRT promoted greater increases in the pennation angle compared to both CON and FWRT. Eccentric resistance training has been reported to cause increases in FL, and initial evidence has subsequently indicated that hip‐dominant FWRT increases FL in the biceps femoris muscle (Timmins et al., 2021). In contrast, concentric or conventional concentric‐eccentric exercise training has been shown to increase the pennation angle (Franchi et al., 2014; Reeves et al., 2009), which likely explains the unique increase in the pennation angle observed in the TRT group in the present study. As described above, we also observed region‐specific VL hypertrophy following FWRT versus TRT. These findings support and extend the findings of Franchii and colleagues (Franchi et al., 2014), who reported greater increases in FL and distal muscle belly hypertrophy following eccentric resistance training but greater increases in the pennation angle and mid‐belly hypertrophy following concentric resistance training. It has been hypothesized that these preferential increases in FL versus pennation angle reflect differential addition of sarcomeres in series versus in parallel in response to eccentric versus conventional concentric–eccentric resistance training (Franchi et al., 2017) and thus that these unique structural adaptations may explain the contraction‐type specific regional hypertrophy as observed both in the present study and in the previous study of (Franchi et al., 2014).

Both TRT and FWRT similarly increased MVIT, which was the only strength measure nonspecific to the exercise modalities in the current study (Table 3). Further, TRT and FWRT elicited mode‐specific strength improvements in isotonic versus flywheel exercise. More specifically, only TRT caused improvements in free weight bench press 1RM. Further, while both TRT and FWRT significantly increased free weight squat 1RM, TRT did so to a greater extent than FWRT. In contrast, only FWRT increased 3W_max_ in the bench press and squat on the flywheel device. The similar increases in nonspecific isometric strength observed in the current study agree with prior data comparing flywheel to traditional training. For example, Caruso et al. reported similar improvements in leg extension concentric and eccentric peak torque at 4.86 rad/s following 10 weeks of leg press training using either a flywheel device or a plate‐loaded leg press machine (Caruso et al., 2005). Similarly, Norrbrand et al. (2008) reported that individuals engaging in knee extension training using a flywheel device or a weight stack machine for 5 weeks similarly improved their isometric knee extension torque by ∼5%–8%. Our data add to these prior studies and further indicate that each resistance exercise modality elicits strength improvements specific to the modality, although it is notable that FWRT also significantly improved free weight squat 1RM but to a lesser degree than TRT.

There is a well‐established association of fiber length with shortening velocity (Bodine et al., 1982; Cramer et al., 2017; Josephson, 1975). Thus, increases in FL by serial sarcomerogenesis may have profound effects on skeletal muscle performance by improving maximal shortening velocity (Franchi et al., 2017). Our findings indicated that FWRT uniquely increased countermovement and broad jump performance compared to CON. The greater jump performance derived from flywheel training in the current study agrees with some (Maroto‐Izquierdo, García‐López, & de Paz, 2017; Stojanović et al., 2021), but not all prior studies (de et al., 2015). Furthermore, we observed moderate relations between the change in VL FL and the change in both broad (*r* = 0.49) and countermovement jump (*r* = 0.35) performance in the present study. Therefore, our data provide evidence to support that FWRT promotes unique improvements in high‐velocity muscle performance in association with increased FL and presumably increases in shortening velocity. Future studies will be necessary to characterize the effects of FWRT on the force–velocity relationship and maximal shortening velocity in humans more fully.

There are several limitations of the present that we would like to acknowledge. Ideally, we would have tested both eccentric and concentric strength across a velocity continuum, but we were only able to test isometric and dynamic strength due to equipment limitations. While the flywheel device in the current study recorded movement velocity, we did not record movement velocity in TRT. Therefore, the FWRT group may have executed repetitions at a faster velocity due to the feedback provided by the device. However, all participants in the TRT group were instructed and encouraged to perform each repetition as explosively as possible. Finally, while we were powered to detect the group × visit interaction for muscle size and function, it is also likely that we were not sufficiently powered for all comparisons examined herein, and we were also not powered to examine sex‐specific effects. Future studies are needed to address these limitations.

In summary, we show that compared to a non‐exercise control, a 10‐week, volume‐matched, total‐body TRT, and FWRT program significantly increased maximal isometric strength and skeletal muscle size. Further, TRT and FWRT caused strength improvements during free weight and flywheel exercise in a mode‐specific manner such that TRT caused the greatest improvements in free weight squat and bench 1RM, whereas FWRT augmented flywheel squat and bench 3W_max_ performance. Notably, FWRT also promoted unique skeletal muscle architectural adaptations that likely contributed to region‐specific hypertrophy and performance improvements. Thus, in the present study, FWRT provided a novel training stimulus that promotes architectural adaptations that support improved athletic performance in a manner that is not provided by traditional resistance exercise training. Future studies should investigate the importance of when the braking window occurs during the eccentric phase of movements (i.e., first‐third vs. last‐third), if diversifying this window during a training program is beneficial, and if there are potential consequences of limiting the eccentric stimulus to a specific window versus evenly across the entire eccentric range of motion.

## AUTHOR CONTRIBUTION

The study was conceptualized and planned by NFB and NDMJ. Data collection and entry were completed by NFB, EMR, and ACB. Data analysis was performed by NFB and NDMJ. NFB was the primary creator of all figures and tables. NFB and NDMJ completed the original draft, and all authors contributed to the review and editing. NFB was responsible for project administration and provided resources to complete the project. All authors approved the final version of the manuscript and agree to be accountable for all aspects of the work in ensuring that questions related to the accuracy or integrity of any part of the work are appropriately investigated and resolved. All persons designated as authors qualify for authorship, and all those who qualify for authorship are listed.

## CONFLICT OF INTEREST STATEMENT

Within the last 3 years, NFB and NFB have received graduate assistant stipend funding from Woodbolt, LLC. NFB and NDMJ have received grant funding from the National Strength and Conditioning Association. EMR has received grant funding from the American College of Sports Medicine. NDMJ has received grant funding from the American Heart Association, the Center for Integrative Research on Childhood Adversity (Award P20GM109097 through the NIGMS), the Injury Prevention Research Center (Award R49 CE003095 through the NCIPC/CDC), the National Institutes of Aging through the Research Network on Animal Models to Understand Social Dimensions of Aging, Woodbolt Distribution, LLC, and Applied Food Sciences, Inc, and has been the recipient of an NIH Clinical Research Loan Repayment Award. The results of the study are presented clearly, honestly, and without fabrication, falsification, or inappropriate data manipulation.
